# Structural data of structure variation and luminescence of 3D, 2D and 1D lanthanide coordination polymers with 1,3-adamantanediacetic acid

**DOI:** 10.1016/j.dib.2018.08.148

**Published:** 2018-09-08

**Authors:** Cheng-Hui Zeng, Kai Zheng, Hao-Ran Li, Zhi-Peng Zhao, Shengliang Zhong, Ye-Fei Jiang

**Affiliations:** College of Chemistry and Chemical Engineering, Key Laboratory of Functional Small Organic Molecule, Ministry of Education and Jiangxi׳s Key Laboratory of Green Chemistry, Jiangxi Normal University, Nanchang 330022, PR China

## Abstract

In this data article, we report the structure, Fourier transform infrared spectroscopy(FT-IR), powder X-ray diffraction (PARD), luminescence decay, thermogravimetric analysis (TGA) and UV–vis data of three series Ln-MOFs. Detailed structure and luminescence properties were discussed in our previous study (Zhao et al., 2018) [1]. The data includes the structure patterns of ligand H_2_ADA, FT-IR, PXRD and thermostability of Ln-MOFs in the air, detailed structure information for these structures are listed in Table 1, Table 2, Table 3, Table 4, Table 5, Table 6, Table 7.

**Specifications table**

**Table:** 

Subject area	*Chemistry*
More specific subject area	*Single crystal data of lanthanide complexes*
Type of data	*Table, figure*
How data was acquired	*Crystallography open data base and crystallographic tool – Diamond: Crystallographic Information File Code: 1562086–1562091 1574790.cif*
Data format	*Analyzed*
Experimental factors	*Single crystal X-ray diffraction data was collected on a Bruker SMART 1000 CCD at 293 K, with Mo-Ka radiation (0.71073 Å). The structure was refined by full-matrix least-squares methods with SHELXL-97 module. The three series structures crystalize in orthorhombic space group Pna2*_*1*_*(no. 33), triclinic space group P-1 (no. 2) and monoclinic space group P2(1)/n.*
Experimental features	*Block colorless single crystals.*
Data source location	*Jiangxi Normal University, Nanchang, China.*
Data accessibility	*The data are with this article.*
Related research article	Zhi-Peng Zhao,^*a*^ Kai Zheng,^*a*^ Hao-Ran Li,^*a*^ Cheng-Hui Zeng,^a,b,d^* Shengliang Zhong,^a^* Seik Weng Ng,^*d*^ Yanqiong Zheng,^*d*^ Yun Chen^*a*^*,* Structure Variation and Luminescence of 3D, 2D and 1D Lanthanide Coordination Polymers with 1,3-Adamantanediacetic Acid, *Inorganica Chimica Acta, revised.*

**Value of the data**

• This data would be valuable for other properties studies of lanthanide complexes that based on 1,3-Adamantanediacetic Acid.
• This data would be valuable for synthesizing lanthanide complexes that coordinated by dmp.
• This data provide a new strategy to control the structure of lanthanide complexes.

## 1 Data

**Fig. 1:**
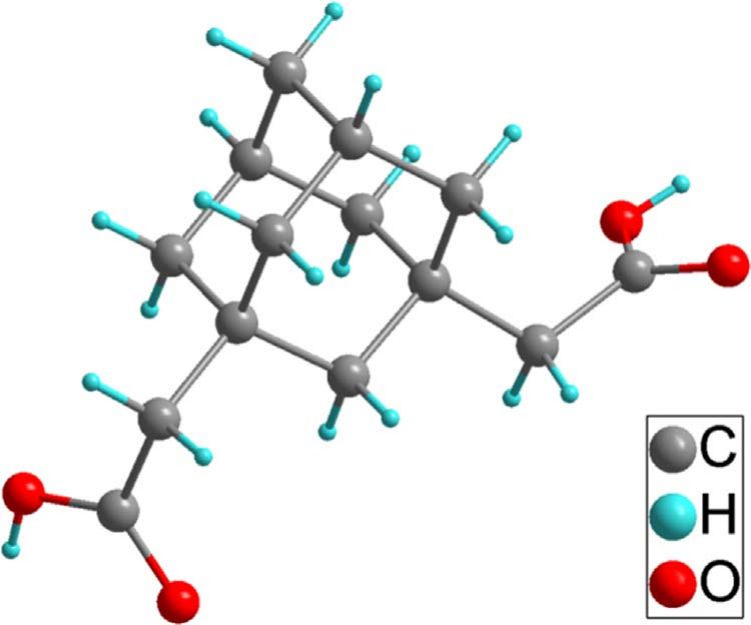
Structure of the ligand H_2_ADA.

**Fig. 2:**
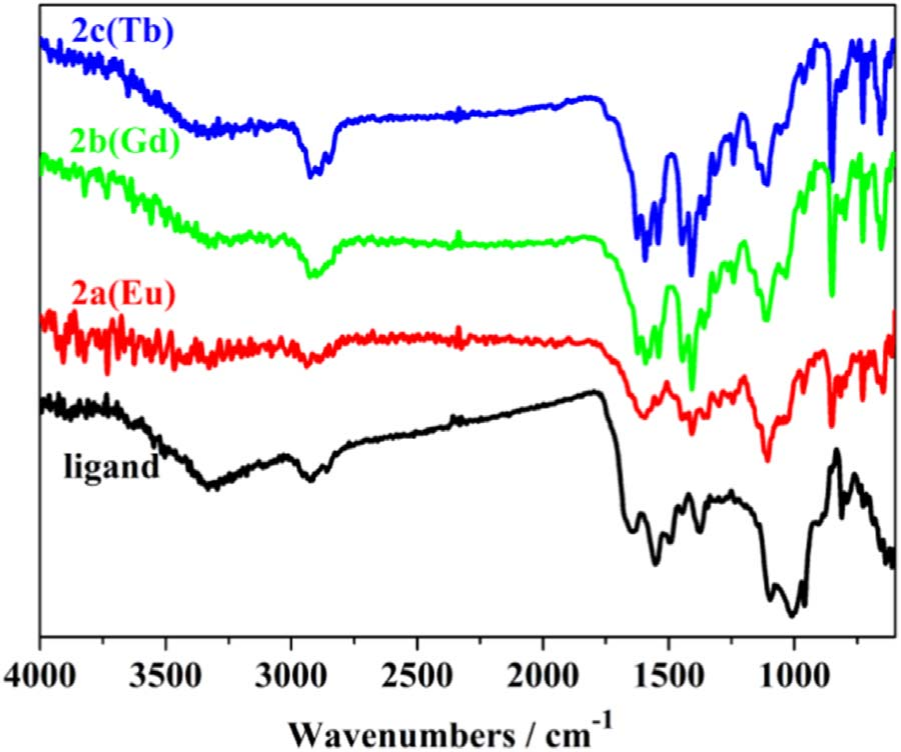
FT-IR spectra of the ligand and **2a**-2**c**.

**Fig. 3:**
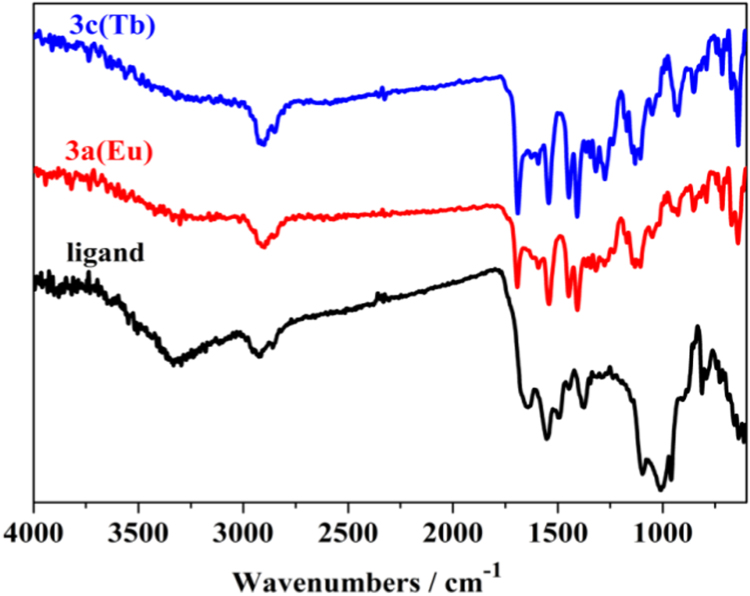
FT-IR spectra of the ligand, **3a** and 3**c**.

**Fig. 4:**
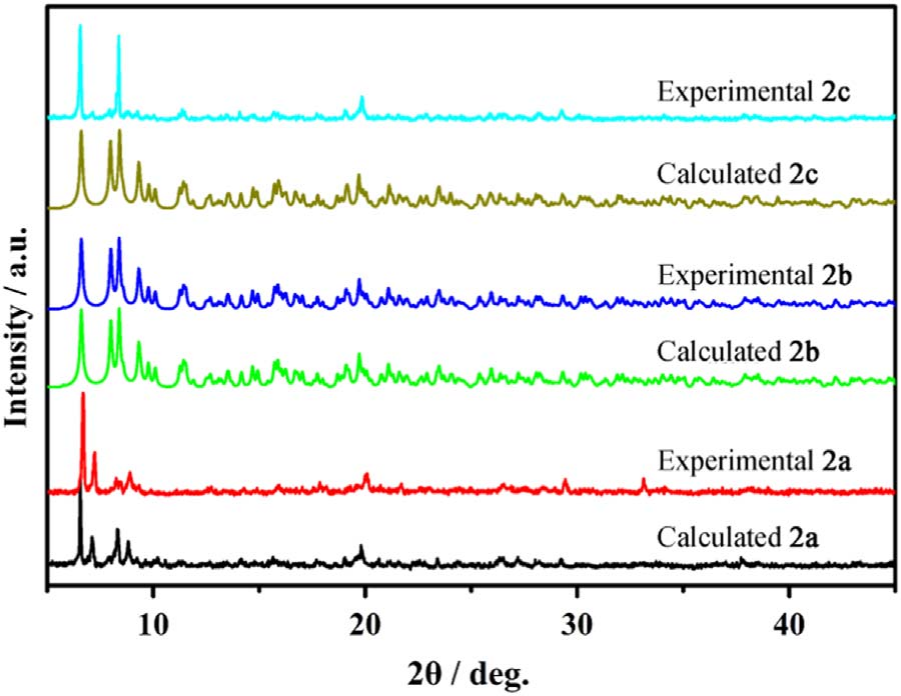
Experimental and calculated PXRD of **2a**, **2b** and **2c** indicate phase purity of the as-synthesized samples.

**Fig. 5:**
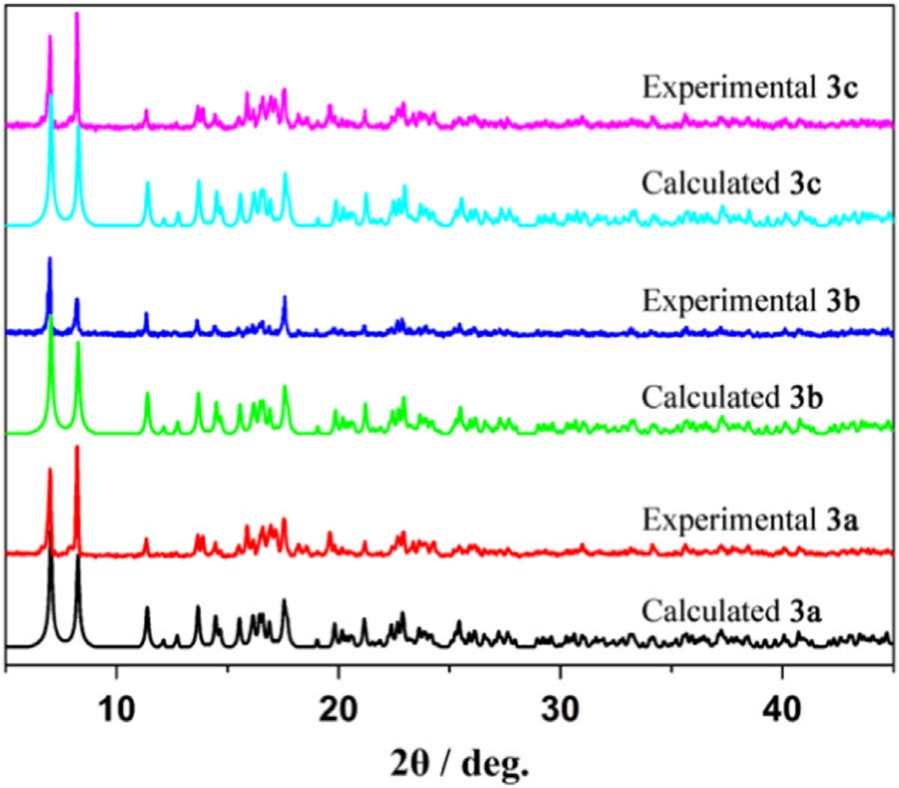
PXRD patterns of bulk samples **3a**, **3b** and **3c** compete well with their simulated results of **3a**, **3b** and **3c**, indicating the high phase purity of bulk samples **3a**, **3b** and **3c**.

**Fig. 6:**
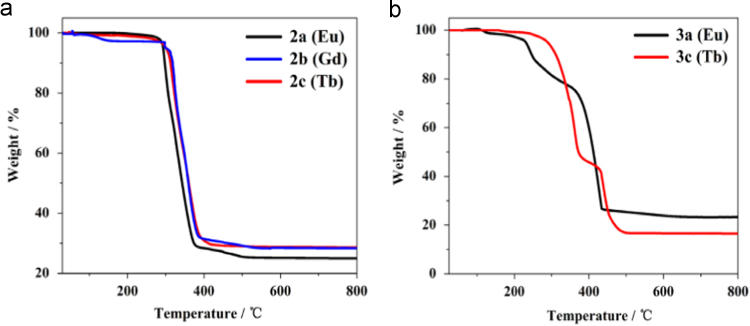
a) TGA of 2D Ln-CPs **2a**, **2b** and **2c** in the air atmosphere; b) TGA of 1D Ln-CPs of **3a** and **3c** in the air atmosphere.

**Fig. 7:**
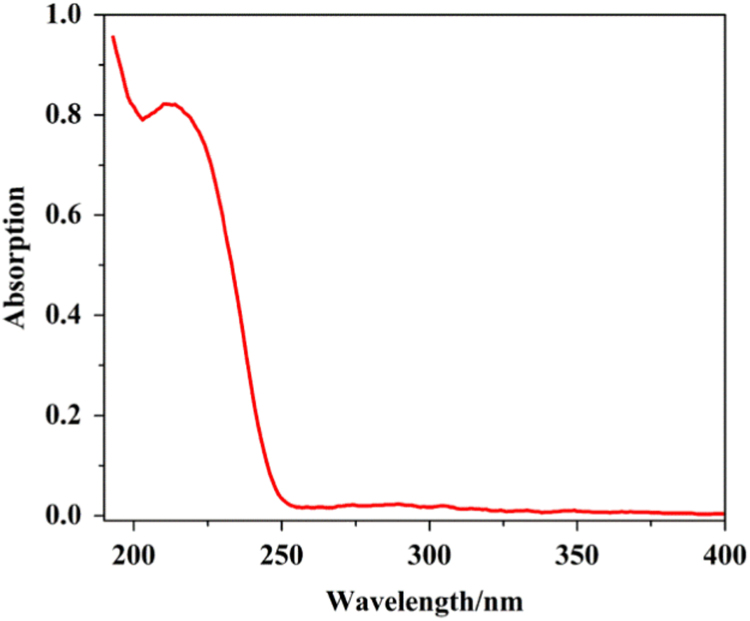
UV–vis spectrum of solid state sample H_2_ADA at room temperature.

Three series of lanthanide coordination polymers (Ln-CPs) [1], 3D Ln-CPs [Tb_2_(ADA)_3_]_n_ (**1a**, H_2_ADA = 1,3-Adamantane-diacetic Acid), 2D Ln-CPs [Ln_2_(ADA)_3_(dmp)_2_]_n_·2EtOH·H_2_O, (Ln^3+^ = Eu^3+^, **2a**; Gd^3+^, **2b**; Tb^3+^, **2c**; dmp = 4,7-dimethyl-1,10-phenanthroline), and 1D Ln-CPs [Ln(ADA)(HADA)(H_2_O)]_n_ (Ln^3+^ = Eu^3+^, **3a**; Gd^3+^, **3b**; Tb^3+^, **3c**), by using the ligand H_2_ADA (Fig. 1). The Ln-CPs are characterized by single-crystal X-ray diffraction, FT-IR (Fig. 2, Fig. 3), PXRD (Fig. 4, Fig. 5), TGA (Fig. 6) and UV–vis (Fig. 7). Detailed information about selected bond lengths and angles for 1a, 2a–2c and 3a–3c are listed in Table 1, Table 2, Table 3, Table 4, Table 5, Table 6, Table 7, they show that the bond lengths and angles are in the normal value as known lanthanide complexes [2], [3], [4], [5], [6], [7].

**Table 1:** Selected bond lengths and bond angles of **1a**.

Tb(1)-O(1)	2.248(6)	Tb(2)-O(2)	2.301(5)
Tb(1)-O(3)	2.263(6)	Tb(2)-O(4)	2.283(6)
Tb(1)-O(5)	2.267(5)	Tb(2)O(6)	2.260(5)
Tb(1)-O(7)	2.325(4)	Tb(2)-O(7)	2.551(4)
Tb(1)-O(9)	2.331(5)	Tb(2)-O(8)	2.432(5)
Tb(1)-O(11)	2.562(5)	Tb(2)-O(9)	2.547(5)
Tb(1)-O(12)	2.389(4)	Tb(2)-O(10)	2.414(5)
Tb(1)-Tb(2)	3.9029(5)	Tb(2)-O(11)	2.344(5)
O(1)-Tb(1)-O(3)	173.8(2)	O(6)-Tb(2)-O(10)	127.94(18)
O(1)-Tb(1)-O(5)	91.9(2)	O(4)-Tb(2)-O(10)	84.07(19)
O(3)-Tb(1)-O(5)	93.2(2)	O(2)-Tb(2)-O(10)	75.9(2)
O(1)-Tb(1)-O(7)	86.05(19)	O(11)-Tb(2)-O(10)	75.76(16)
O(3)-Tb(1)-O(7)	91.0(2)	O(6)-Tb(2)-O(8)	76.07(19)
O(5)-Tb(1)-O(7)	82.02(18)	O(4)-Tb(2)-O(8)	74.86(19)
O(1)-Tb(1)-O(9)	91.4(2)	O(2)-Tb(2)-O(8)	127.2(2)
O(3)-Tb(1)-O(9)	93.0(2)	O(11)-Tb(2)-O(8)	87.63(18)
O(5)-Tb(1)-O(9)	81.18(18)	O(10)-Tb(2)-O(8)	151.56(18)
O(7)-Tb(1)-O(9)	162.91(15)	O(6)-Tb(2)-O(9)	78.02(17)
O(1)-Tb(1)-O(12)	90.1(2)	O(4)-Tb(2)-O(9)	79.77(19)
O(3)-Tb(1)-O(12)	86.6(2)	O(2)-Tb(2)-O(9)	80.38(19)
O(5)-Tb(1)-O(12)	158.2(2)	O(11)-Tb(2)-O(9)	127.76(15)
O(7)-Tb(1)-O(12)	119.83(18)	O(10)-Tb(2)-O(9)	52.00(14)
O(9)-Tb(1)-O(12)	77.02(18)	O(8)-Tb(2)-O(9)	138.53(17)
O(1)-Tb(1)-O(11)	89.3(2)	O(6)-Tb(2)-O(7)	83.50(17)
O(3)-Tb(1)-O(11)	84.5(2)	O(4)-Tb(2)-O(7)	123.67(18)
O(5)-Tb(1)-O(11)	149.43(18)	O(2)-Tb(2)-O(7)	78.08(19)
O(7)-Tb(1)-O(11)	67.58(16)	O(11)-Tb(2)-O(7)	67.51(15)
O(9)-Tb(1)-O(11)	129.35(15)	O(10)-Tb(2)-O(7)	135.23(16)
O(12)-Tb(1)-O(11)	52.33(17)	O(8)-Tb(2)-O(7)	51.96(16)
O(1)-Tb(1)-Tb(2)	70.94(16)	O(9)-Tb(2)-O(7)	153.07(15)
O(3)-Tb(1)-Tb(2)	103.41(19)	O(6)-Tb(2)-Tb(1)	112.78(15)
O(5)-Tb(1)-Tb(2)	117.52(14)	O(4)-Tb(2)-Tb(1)	129.17(17)
O(7)-Tb(1)-Tb(2)	38.86(11)	O(2)-Tb(2)-Tb(1)	65.19(16)
O(9)-Tb(1)-Tb(2)	153.79(11)	O(11)-Tb(2)-Tb(1)	39.29(12)
O(12)-Tb(1)-Tb(2)	83.62(15)	O(10)-Tb(2)-Tb(1)	100.58(12)
O(11)-Tb(1)-Tb(2)	35.39(10)	O(8)-Tb(2)-Tb(1)	79.19(13)
O(6)-Tb(2)-O(4)	102.3(2)	O(9)-Tb(2)-Tb(1)	141.34(11)
O(6)-Tb(2)-O(2)	83.1(2)	O(7)-Tb(2)-Tb(1)	34.87(10)
O(4)-Tb(2)-O(2)	157.8(2)	O(4)-Tb(2)-O(11)	96.3(2)
O(6)-Tb(2)-O(11)	150.87(19)	O(2)-Tb(2)-O(11)	88.1(2)

**Table 2:** Selected bond lengths and bond angles of **2a**.

Eu(1)-O(8)#1	2.358(3)	Eu(2)-O(6)#4	2.328(3)
Eu(1)-O(12)#2	2.381(3)	Eu(2)-O(1)	2.337(3)
Eu(1)-O(11)#3	2.379(3)	Eu(2)-O(2)#4	2.344(3)
Eu(1)-O(10)	2.427(3)	Eu(2)-O(5)	2.365(3)
Eu(1)-O(7)	2.510(3)	Eu(2)-O(3)#5	2.449(3)
Eu(1)-N(4)	2.558(4)	Eu(2)-O(4)#5	2.516(3)
Eu(1)-O(8)	2.564(3)	Eu(2)-N(2)	2.606(4)
Eu(1)-O(9)	2.587(3)	Eu(2)-N(1)	2.627(4)
Eu(1)-N(3)	2.652(4)	Eu(1)-Eu(1)#1	3.9343(4)
O(8)#1-Eu(1)-O(12)#2	77.01(10)	O(4)#5-Eu(2)-N(1)	71.36(11)
O(8)#1-Eu(1)-O(11)#3	74.13(10)	N(2)-Eu(2)-N(1)	61.85(11)78.29(11)
O(12)#2-Eu(1)-O(11)#3	137.39(9)	O(1)-Eu(2)-O(3)#5	144.66(10)
O(8)#1-Eu(1)-O(10)	88.56(11)	O(2)#4-Eu(2)-O(3)#5	134.71(10)
O(12)#2-Eu(1)-O(10)	128.82(10)	O(5)-Eu(2)-O(3)#5	136.07(10)
O(11)#3-Eu(1)-O(10)	81.14(10)	O(6)#4-Eu(2)-O(4)#5	80.20(11)
O(8)#1-Eu(1)-O(7)	124.75(9)	O(1)-Eu(2)-O(4)#5	145.06(10)
O(12)#2-Eu(1)-O(7)	80.55(10)	O(2)#4-Eu(2)-O(4)#5	85.99(10)
O(11)#3-Eu(1)-O(7)	90.85(10)	O(5)-Eu(2)-O(4)#5	52.32(10)
O(10)-Eu(1)-O(7)	142.24(11)	O(3)#5-Eu(2)-O(4)#5	83.57(11)
O(8)#1-Eu(1)-N(4)	144.91(11)	O(6)#4-Eu(2)-N(2)	144.79(11)
O(12)#2-Eu(1)-N(4)	80.23(11)	O(1)-Eu(2)-N(2)	77.67(11)
O(11)#3-Eu(1)-N(4)	138.26(11)	O(2)#4-Eu(2)-N(2)	136.36(11)
O(10)-Eu(1)-N(4)	85.25(11)	O(5)-Eu(2)-N(2)	142.67(9)
O(7)-Eu(1)-N(4)	76.45(11)	O(8)-Eu(1)-O(9)	145.73(11)
O(8)#1-Eu(1)-O(8)	73.92(10)	O(8)#1-Eu(1)-N(3)	137.23(11)
O(12)#2-Eu(1)-O(8)	70.60(9)	O(12)#2-Eu(1)-N(3)	75.83(11)
O(11)#3-Eu(1)-O(8)	71.70(9)	O(11)#3-Eu(1)-N(3)	70.79(12)
O(10)-Eu(1)-O(8)	150.73(10)	O(10)-Eu(1)-N(3)	71.47(11)
O(7)-Eu(1)-O(8)	51.02(9)	O(7)-Eu(1)-N(3)	62.43(12)
N(4)-Eu(1)-O(8)	122.43(10)	N(4)-Eu(1)-N(3)	111.47(10)
O(8)#1-Eu(1)-O(9)	80.38(10)	O(8)-Eu(1)-N(3)	105.06(11)
O(12)#2-Eu(1)-O(9)	77.63(9)	O(9)-Eu(1)-N(3)	78.91(11)
O(11)#3-Eu(1)-O(9)	126.33(9)	O(6)#4-Eu(2)-O(1)	76.54(10)
O(10)-Eu(1)-O(9)	51.45(10)	O(6)#4-Eu(2)-O(2)#4	126.31(11)
O(7)-Eu(1)-O(9)	141.46(10)	O(1)-Eu(2)-O(2)#4	125.92(11)
N(4)-Eu(1)-O(9)	68.83(11)	O(6)#4-Eu(2)-O(5)	77.74(11)
O(2)#4-Eu(2)-O(5)	79.43(10)	O(1)-Eu(2)-O(5)	
O(6)#4-Eu(2)-O(3)#5	85.68(11)		

Symmetry transformations used to generate equivalent atoms: #1 −*x*+2, −*y*, −*z*+1; # 2 *x*+1, *y*, *z*; #3 −*x*+1, −y, −*z*+1; #4 −*x*+2, −*y*−1, −*z*; #5 −*x*+3, −*y*−1, −*z*; #6 *x*−1, *y*, *z*.

**Table 3:** Selected bond lengths and bond angles of **2b**.

Gd(1)-O(8)#1	2.341(3)	Gd(1)-Gd(1)#1	3.9190(3)
Gd(1)-O(12)#2	2.367(3)	Gd(2)-O(6)#4	2.320(3)
Gd(1)-O(11)#3	2.372(3)	Gd(2)-O(1)	2.326(3)
Gd(1)-O(10)	2.418(3)	Gd(2)-O(2)#4	2.332(3)
Gd(1)-O(7)	2.494(3)	Gd(2)-O(5)	2.348(3)
Gd(1)-N(1)	2.546(3)	Gd(2)-O(3)#5	2.440(3)
Gd(1)-O(8)	2.560(2)	Gd(2)-O(4)#5	2.504(3)
Gd(1)-O(9)	2.584(3)	Gd(2)-N(4)	2.591(3)
Gd(1)-N(2)	2.641(4)	Gd(2)-N(3)	2.610(3)
O(8)#1-Gd(1)-O(12)#2	74.36(9)	O(6)#4-Gd(2)-O(1)	78.93(10)
O(8)#1-Gd(1)-O(11)#3	77.01(9)	O(6)#4-Gd(2)-O(2)#4	76.77(10)
O(12)#2-Gd(1)-O(11)#3	137.46(8)	O(1)-Gd(2)-O(2)#4	126.07(10)
O(8)#1-Gd(1)-O(10)	88.69(10)	O(6)#4-Gd(2)-O(5)	125.63(11)
O(12)#2-Gd(1)-O(10)	81.10(9)	O(1)-Gd(2)-O(5)	77.43(10)
O(11)#3-Gd(1)-O(10)	129.04(10)	O(2)#4-Gd(2)-O(5)	79.05(10)
O(8)#1-Gd(1)-O(7)	124.92(8)	O(6)#4-Gd(2)-O(3)#5	85.25(10)
O(12)#2-Gd(1)-O(7)	90.75(9)	O(1)-Gd(2)-O(3)#5	78.23(10)
O(11)#3-Gd(1)-O(7)	80.56(10)	O(2)#4-Gd(2)-O(3)#5	144.87(10)
O(10)-Gd(1)-O(7)	141.94(10)	O(5)-Gd(2)-O(3)#5	135.05(9)
O(8)#1-Gd(1)-N(1)	144.54(11)	O(6)#4-Gd(2)-O(4)#5	135.86(10)
O(12)#2-Gd(1)-N(1)	138.45(11)	O(1)-Gd(2)-O(4)#5	80.13(10)
O(11)#3-Gd(1)-N(1)	79.90(10)	O(2)#4-Gd(2)-O(4)#5	145.12(10)
O(10)-Gd(1)-N(1)	85.31(10)	O(5)-Gd(2)-O(4)#5	86.32(10)
O(7)-Gd(1)-N(1)	76.44(10)	O(3)#5-Gd(2)-O(4)#5	52.53(10)
O(8)#1-Gd(1)-O(8)	73.91(10)	O(6)#4-Gd(2)-N(4)	83.38(10)
O(12)#2-Gd(1)-O(8)	71.58(8)	O(1)-Gd(2)-N(4)	144.84(11)
O(11)#3-Gd(1)-O(8)	70.66(8)	O(2)#4-Gd(2)-N(4)	77.88(10)
O(10)-Gd(1)-O(8)	150.61(9)	O(5)-Gd(2)-N(4)	136.72(10)
O(7)-Gd(1)-O(8)	51.21(8)	O(3)#5-Gd(2)-N(4)	70.17(10)
N(1)-Gd(1)-O(8)	122.49(9)	O(4)#5-Gd(2)-N(4)	92.22(10)
O(8)#1-Gd(1)-O(9)	80.14(9)	O(6)#4-Gd(2)-N(3)	138.83(10)
O(12)#2-Gd(1)-O(9)	126.29(9)	O(1)-Gd(2)-N(3)	142.21(10)
O(11)#3-Gd(1)-O(9)	77.71(9)	O(2)#4-Gd(2)-N(3)	74.53(10)
O(10)-Gd(1)-O(9)	51.55(9)	O(5)-Gd(2)-N(3)	76.55(10)
O(7)-Gd(1)-O(9)	141.57(9)	O(3)#5-Gd(2)-N(3)	102.01(10)
N(1)-Gd(1)-O(9)	68.83(10)	O(4)#5-Gd(2)-N(3)	71.31(10)
O(8)-Gd(1)-O(9)	142.65(9)	N(4)-Gd(2)-N(3)	62.23(10)
O(8)#1-Gd(1)-N(2)	146.01(10)	O(7)-Gd(1)-N(2)	71.20(10)
O(12)#2-Gd(1)-N(2)	75.87(10)	N(1)-Gd(1)-N(2)	62.57(11)
O(11)#3-Gd(1)-N(2)	136.95(10)	O(8)-Gd(1)-N(2)	111.41(10)

Symmetry transformations used to generate equivalent atoms: #1 −*x*+1, −*y*+2, −*z*; #2 −*x*+2, −*y*+2, −*z*; #3 *x*−1, *y*, *z*; #4 −*x*+1, −*y*+3, −*z*+1; #5 −*x*, −*y*+3, −*z*+1; #6 *x*+1, *y*, *z*.

**Table 4:** Selected bond lengths and bond angles of **2c**.

Tb(1)-O(2)#1	2.335(2)	Tb(1)-Tb(1)#1	3.9023(3)
Tb(1)-O(6)#1	2.352(2)	Tb(2)-O(3)	2.300(3)
Tb(1)-O(5)	2.354(2)	Tb(2)-O(10)	2.310(3)
Tb(1)-O(7)#2	2.400(3)	Tb(2)-O(9)#3	2.313(2)
Tb(1)-O(1)	2.484(3)	Tb(2)-O(4)#3	2.337(3)
Tb(1)-N(1)	2.525(3)	Tb(2)-O(11)#4	2.425(3)
Tb(1)-O(2)	2.550(2)	Tb(2)-O(12)#4	2.492(3)
Tb(1)-O(8)#2	2.587(3)	Tb(2)-N(3)	2.583(3)
Tb(1)-N(2)	2.624(3)	Tb(2)-N(4)	2.590(3)
O(2)#1-Tb(1)-O(6)#1	74.52(9)	O(3)-Tb(2)-O(10)	79.03(10)
O(2)#1-Tb(1)-O(5)	77.01(9)	O(3)-Tb(2)-O(9)#3	77.05(9)
O(6)#1-Tb(1)-O(5)	137.73(8)	O(10)-Tb(2)-O(9)#3	125.97(10)
O(2)#1-Tb(1)-O(7)#2	88.32(10)	O(3)-Tb(2)-O(4)#3	125.84(10)
O(6)#1-Tb(1)-O(7)#2	80.97(9)	O(10)-Tb(2)-O(4)#3	77.41(10)
O(5)-Tb(1)-O(7)#2	128.74(9)	O(9)#3-Tb(2)-O(4)#3	78.78(9)
O(2)#1-Tb(1)-O(1)	125.17(8)	O(3)-Tb(2)-O(11)#4	84.63(10)
O(6)#1-Tb(1)-O(1)	91.05(9)	O(10)-Tb(2)-O(11)#4	78.30(10)
O(5)-Tb(1)-O(1)	80.51(9)	O(9)#3-Tb(2)-O(11)#4	144.78(9
O(7)#2-Tb(1)-O(1)	142.22(10)	O(4)#3-Tb(2)-O(11)#4	135.47(9)
O(2)#1-Tb(1)-N(1)	144.25(10)	O(3)-Tb(2)-O(12)#4	135.52(9)
O(6)#1-Tb(1)-N(1)	138.48(10)	O(10)-Tb(2)-O(12)#4	80.05(10)
O(5)-Tb(1)-N(1)	79.71(10)	O(9)#3-Tb(2)-O(12)#4	145.18(9)
O(7)#2-Tb(1)-N(1)	85.47(10)	O(4)#3-Tb(2)-O(12)#4	86.50(10)
O(1)-Tb(1)-N(1)	76.33(9)	O(11)#4-Tb(2)-O(12)#4	52.79(9)
O(2)#1-Tb(1)-O(2)	74.05(9)	O(3)-Tb(2)-N(3)	82.85(10)
O(6)#1-Tb(1)-O(2)	71.62(8)	O(10)-Tb(2)-N(3)	144.69(10)
O(5)-Tb(1)-O(2)	70.91(8)	O(9)#3-Tb(2)-N(3)	77.99(10)
O(7)#2-Tb(1)-O(2)	150.42(9)	O(4)#3-Tb(2)-N(3)	136.97(9)
O(1)-Tb(1)-O(2)	51.36(8)	O(11)#4-Tb(2)-N(3)	70.00(9)
N(1)-Tb(1)-O(2)	122.59(9)	O(12)#4-Tb(2)-N(3)	92.45(10)
O(2)#1-Tb(1)-O(8)#2	79.78(8)	O(3)-Tb(2)-N(4)	138.85(10)
O(6)#1-Tb(1)-O(8)#2	126.31(9)	O(10)-Tb(2)-N(4)	142.10(10)
O(5)-Tb(1)-O(8)#2	77.30(8)	O(9)#3-Tb(2)-N(4)	74.65(9)
O(7)#2-Tb(1)-O(8)#2	51.67(9)	O(4)#3-Tb(2)-N(4)	76.60(10)
O(1)-Tb(1)-O(8)#2	141.32(8)	O(11)#4-Tb(2)-N(4)	102.10(10)
N(1)-Tb(1)-O(8)#2	68.84(10)	O(12)#4-Tb(2)-N(4)	71.29(9)
O(2)-Tb(1)-O(8)#2	142.41(8)	N(3)-Tb(2)-N(4)	62.54(10)
O(2)#1-Tb(1)-N(2)	145.73(10)	N(1)-Tb(1)-N(2)	63.07(11)
O(6)#1-Tb(1)-N(2)	75.42(10)	O(2)-Tb(1)-N(2)	111.19(9)
O(5)-Tb(1)-N(2)	137.23(10)	O(8)#2-Tb(1)-N(2)	105.68(10)

Symmetry transformations used to generate equivalent atoms: #1 −*x*+1, −*y*+2, −*z*; #2 *x*+1, *y*, *z*; #3 −*x*+1, −*y*+3, −*z*+1; #4 −*x*+2, −*y*+3, −*z*+1; #5 *x*−1, *y*, *z*.

**Table 5:** Selected bond lengths and bond angles of **3a**.

Eu(1)-O(6)#1	2.296(3)	Eu(1)-O(2)	2.610(2)
Eu(1)-O(5)	2.370(2)	Eu(1)-Eu(1)#2	4.1260(3)
Eu(1)-O(2)#2	2.377(2)	Eu(1)-O(4)#1	2.488(2)
Eu(1)-O(9)	2.377(2)	Eu(1)-O(1)	2.421(2)
Eu(1)-O(3)#1	2.415(2)		
O(6)#1-Eu(1)-O(5)	98.94(9)	O(3)#1-Eu(1)-O(1)	155.20(8)
O(6)#1-Eu(1)-O(2)#2	165.00(9)	O(6)#1-Eu(1)-O(4)#1	78.02(9)
O(5)-Eu(1)-O(2)#2	80.39(8)	O(5)-Eu(1)-O(4)#1	134.42(8)
O(6)#1-Eu(1)-O(9)	108.01(9)	O(2)#2-Eu(1)-O(4)#1	91.69(8)
O(5)-Eu(1)-O(9)	145.34(8)	O(9)-Eu(1)-O(4)#1	73.82(7)
O(2)#2-Eu(1)-O(9)	78.98(8)	O(3)#1-Eu(1)-O(4)#1	53.17(8)
O(6)#1-Eu(1)-O(3)#1	84.76(9)	O(1)-Eu(1)-O(4)#1	133.18(8)
O(5)-Eu(1)-O(3)#1	81.27(8)	O(6)#1-Eu(1)-O(2)	125.99(8)
O(2)#2-Eu(1)-O(3)#1	80.32(8)	O(5)-Eu(1)-O(2)	73.93(7)
O(9)-Eu(1)-O(3)#1	121.82(8)	O(2)#2-Eu(1)-O(2)	68.44(8)
O(6)#1-Eu(1)-O(1)	75.15(9)	O(9)-Eu(1)-O(2)	72.69(7)
O(5)-Eu(1)-O(1)	87.55(8)	O(3)#1-Eu(1)-O(2)	142.62(8)
O(2)#2-Eu(1)-O(1)	119.66(8)	O(1)-Eu(1)-O(2)	51.47(7)
O(9)-Eu(1)-O(1)	78.96(8)	O(4)#1-Eu(1)-O(2)	143.66(7)

Symmetry transformations used to generate equivalent atoms: #1 −*x*+2, −*y*+1, −z; #2 −*x*+1, −*y*+1, −*z*.

**Table 6:** Selected bond lengths and bond angles of **3b**.

Gd(1)-O(6)#1	2.2839(19)	Gd(1)-O(3)#1	2.409(2)
Gd(1)-O(5)	2.3557(18)	Gd(1)-O(2)	2.4734(19)
Gd(1)-O(4)#2	2.3658(17)	Gd(1)-O(4)#1	2.5959(18)
Gd(1)-O(1W)	2.3641(18)	Gd(1)-O(1)	2.4069(19)
O(6)#1-Gd(1)-O(5)	98.54(7)	O(1)-Gd(1)-O(3)#1	154.83(7)
O(6)#1-Gd(1)-O(4)#2	164.99(7)	O(6)#1-Gd(1)-O(2)	77.98(7)
O(5)-Gd(1)-O(4)#2	80.77(6)	O(5)-Gd(1)-O(2)	134.30(6)
O(6)#1-Gd(1)-O(1W)	108.48(7)	O(4)#2-Gd(1)-O(2)	91.70(7)
O(5)-Gd(1)-O(1W)	145.49(6)	O(1W)-Gd(1)-O(2)	73.75(6)
O(4)#2-Gd(1)-O(1W)	78.44(7)	O(1)-Gd(1)-O(2)	53.36(6)
O(6)#1-Gd(1)-O(1)	84.59(7)	O(3)#1-Gd(1)-O(2)	133.25(7)
O(5)-Gd(1)-O(1)	80.96(7)	O(6)#1-Gd(1)-O(4)#1	126.04(6)
O(4)#2-Gd(1)-O(1)	80.48(6)	O(5)-Gd(1)-O(4)#1	74.01(6)
O(1W)-Gd(1)-O(1)	121.79(7)	O(4)#2-Gd(1)-O(4)#1	68.38(7)
O(6)#1-Gd(1)-O(3)#1	75.13(7)	O(1W)-Gd(1)-O(4)#1	72.83(6)
O(5)-Gd(1)-O(3)#1	87.38(7)	O(1)-Gd(1)-O(4)#1	142.47(6)
O(4)#2-Gd(1)-O(3)#1	119.69(6)	O(3)#1-Gd(1)-O(4)#1	51.55(6)
O(1W)-Gd(1)-O(3)#1	79.49(7)	O(2)-Gd(1)-O(4)#1	143.82(6)

Symmetry transformations used to generate equivalent atoms: #1 −*x*+1, −*y*+1, −*z*; #2 *x*−1, *y*, *z*; #3 *x*+1, *y*, z.

**Table 7:** Selected bond lengths and bond angles of **3c**.

Tb(1)-O(2)#1	2.266(2)	Tb(1)-O(6)#3	2.394(2)
Tb(1)-O(0AA)	2.3371(19)	Tb(1)-O(8)#2	2.461(2)
Tb(1)-O(5)	2.3472(19)	Tb(1)-O(5)#3	2.597(2)
Tb(1)-O(1W)	2.3457(19)	Tb(1)-O(7)#2	2.390(2)
O(2)#1-Tb(1)-O(0AA)	98.14(8)	O(1W)-Tb(1)-O(6)#3	79.64(8)
O(2)#1-Tb(1)-O(5)	164.70(8)	O(7)#2-Tb(1)-O(6)#3	154.51(7)
O(0AA)-Tb(1)-O(5)	80.94(7)	O(2)#1-Tb(1)-O(8)#2	78.02(8)
O(2)#1-Tb(1)-O(1W)	108.68(8)	O(0AA)-Tb(1)-O(8)#2	134.32(7)
O(0AA)-Tb(1)-O(1W)	145.71(7)	O(5)-Tb(1)-O(8)#2	91.64(7)
O(5)-Tb(1)-O(1W)	78.56(7)	O(1W)-Tb(1)-O(8)#2	73.65(7)
O(2)#1-Tb(1)-O(7)#2	84.43(8)	O(7)#2-Tb(1)-O(8)#2	53.75(7)
O(0AA)-Tb(1)-O(7)#2	80.60(7)	O(6)#3-Tb(1)-O(8)#2	133.26(7)
O(5)-Tb(1)-O(7)#2	80.35(7)	O(2)#1-Tb(1)-O(5)#3	126.18(7)
O(1W)-Tb(1)-O(7)#2	122.08(7)	O(0AA)-Tb(1)-O(5)#3	74.32(6)
O(2)#1-Tb(1)-O(6)#3	75.17(8)	O(5)-Tb(1)-O(5)#3	68.47(8)
O(0AA)-Tb(1)-O(6)#3	87.23(7)	O(1W)-Tb(1)-O(5)#3	72.69(7)
O(5)-Tb(1)-O(6)#3	119.88(7)	O(7)#2-Tb(1)-O(5)#3	142.37(7)

Symmetry transformations used to generate equivalent atoms: #1 −*x*+1, −*y*+1, −*z*; #2 *x*+1, *y*, *z*; #3 −*x*, −*y*+1, −*z*; #4 *x*−1, *y*, *z*.

## 2 Experimental design, materials, and methods

Synthesis of **1a**: In a 250 mL Teflon-lined stainless-steel autoclave, 0.396 mmol H_2_ADA, 0.594 mmol Tb(NO_3_)_3_·6H_2_O and 0.396 mmol 1,10-phenanthroline (phen) were mixed, then 100 mL H_2_O added, and mixed by a magnetic stirrer. The reactants were sealed in the Teflon-lined stainless-steel autoclave and heated at 418 K for 6 days.

Synthesis of **2a–2c**: In a 50 mL beaker, 0.396 mmol H_2_ADA and 20 mL H_2_O were mixed, and the pH adjusted to 6 with 0.1 M NaOH solution. Then the solution was mixed with 20 mL MeOH solution containing 0.27 mmol Ln(NO_3_)_3_·6H_2_O. After that, 0.269 mmol dmp dissolved in 20 mL EtOH solution was added to the mixture. Then it was transferred to a bottle and sealed, allowing the reaction to proceed at 333 K for 72 h.

Synthesis of **3a–3c**: In a 100 mL bottle, 0.396 mmol H_2_ADA was mixed with 0.13 mmol Ln(NO_3_)_3_·6H_2_O. 10 mL DMF and 50 mL H_2_O was added and stirred for 10 min. Then the bottle was sealed and reacted at 333 K for 72 h.

The lanthanide salts were obtained by the procedures as our previous work [8], [9], [10], [11], [12], [13]. H_2_ADA (97.0%) was purchased from Innochem (Beijing, China) and used without any purification. Other chemicals (A.R.) are commercially available and were used as received.

Single crystal X-ray diffraction data was collected on a Bruker SMART 1000 CCD, with Mo-Ka radiation (Wavelength = 0.71073 Å) at room temperature. The structure was refined by full-matrix least-squares methods with SHELXL-97 module. FT-IR was obtained in KBr pellets and recorded on a Nicolet 330 FT-IR spectrometer. TGA was recorded on a Netzsch-Bruker TG-209 unit in the air atmosphere. Luminescence spectra and lifetimes were recorded on an Edinburgh FLS980 at room temperature. Phase purity of bulk sample was determined on a DMAX2200VPC diffractometer, at 30 kV and 30 mA.
